# Genomic patterns resembling *BRCA1*- and *BRCA2*-mutated breast cancers predict benefit of intensified carboplatin-based chemotherapy

**DOI:** 10.1186/bcr3655

**Published:** 2014-05-15

**Authors:** Marieke A Vollebergh, Esther H Lips, Petra M Nederlof, Lodewyk FA Wessels, Jelle Wesseling, Marc J vd Vijver, Elisabeth GE de Vries, Harm van Tinteren, Jos Jonkers, Michael Hauptmann, Sjoerd Rodenhuis, Sabine C Linn

**Affiliations:** 1 Department of Molecular Pathology, the Netherlands Cancer Institute - Antoni van Leeuwenhoek Hospital, Plesmanlaan 121, 1066 CX Amsterdam, The Netherlands; 2 Department of Molecular Carcinogenesis, the Netherlands Cancer Institute -Antoni van Leeuwenhoek Hospital, Plesmanlaan 121, 1066 CX Amsterdam, The Netherlands; 3 Department of Pathology, the Netherlands Cancer Institute - Antoni van Leeuwenhoek Hospital, Plesmanlaan 121, 1066 CX Amsterdam, The Netherlands; 4 Department of Biometrics, the Netherlands Cancer Institute - Antoni van Leeuwenhoek Hospital, Plesmanlaan 121, 1066 CX Amsterdam, The Netherlands; 5 Department of Epidemiology and Biostatistics, the Netherlands Cancer Institute - Antoni van Leeuwenhoek Hospital, Plesmanlaan 121, 1066 CX Amsterdam, The Netherlands; 6 Department of Medical Oncology, the Netherlands Cancer Institute - Antoni van Leeuwenhoek Hospital, Plesmanlaan 121, 1066 CX Amsterdam, The Netherlands; 7 Faculty of Electrical Engineering, Mathematics and Computer Science, Delft University of Technology, Stevinweg 1, 2628 CN Delft, The Netherlands; 8 Department of Pathology, Academic Medical Center, Meibergdreef 9, 1105 AZ Amsterdam, The Netherlands; 9 Department of Medical Oncology, University Medical Center Groningen, Hanzeplein 1, 9713 GZ Groningen, The Netherlands; 10 Department of Pathology, University Medical Center Utrecht, Heidelberglaan 100, 3584 CX Utrecht, The Netherlands

## Abstract

**Introduction:**

*BRCA*-mutated breast cancer cells lack the DNA-repair mechanism homologous recombination that is required for error-free DNA double-strand break (DSB) repair. Homologous recombination deficiency (HRD) may cause hypersensitivity to DNA DSB-inducing agents, such as bifunctional alkylating agents and platinum salts. HRD can be caused by *BRCA* mutations, and by other mechanisms. To identify HRD, studies have focused on triple-negative (TN) breast cancers as these resemble *BRCA1*-mutated breast cancer closely and might also share this hypersensitivity. However, ways to identify HRD in non-*BRCA*-mutated, estrogen receptor (ER)-positive breast cancers have remained elusive. The current study provides evidence that genomic patterns resembling *BRCA1-* or *BRCA2*-mutated breast cancers can identify breast cancer patients with TN as well as ER-positive, HER2-negative tumors that are sensitive to intensified, DSB-inducing chemotherapy.

**Methods:**

Array comparative genomic hybridization (aCGH) was used to classify breast cancers. Patients with tumors with similar aCGH patterns as *BRCA1*- and/or *BRCA2*-mutated breast cancers were defined as having a BRCA-like^CGH^ status, others as non-BCRA-like^CGH^. Stage-III patients (n = 249) had participated in a randomized controlled trial of adjuvant high-dose (HD) cyclophosphamide-thiotepa-carboplatin (CTC) versus 5-fluorouracil-epirubicin-cyclophosphamide (FE_90_C) chemotherapy.

**Results:**

Among patients with BRCA-like^CGH^ tumors (81/249, 32%), a significant benefit of HD-CTC compared to FE_90_C was observed regarding overall survival (adjusted hazard ratio 0.19, 95% CI: 0.08 to 0.48) that was not seen for patients with non-BRCA-like^CGH^ tumors (adjusted hazard ratio 0.90, 95% CI: 0.53 to 1.54) (*P* = 0.004). Half of all BRCA-like^CGH^ tumors were ER-positive.

**Conclusions:**

Distinct aCGH patterns differentiated between HER2-negative patients with a markedly improved outcome after adjuvant treatment with an intensified DNA-DSB-inducing regimen (BRCA-like^CGH^ patients) and those without benefit (non-BRCA-like^CGH^ patients).

## Introduction

Adjuvant systemic treatment decisions for early breast cancer are commonly based on results of large randomized clinical trials conducted in the general breast cancer population. Such trials do not take into account the molecular heterogeneity present in breast cancer [1]. Consequently, some treatment strategies that are highly beneficial to a small percentage of the general breast cancer population may have been discarded in the past. An example of such a treatment strategy might be intensified alkylating chemotherapy [2,3]. Here we investigated whether a subgroup of breast cancer patients exists that might derive substantial benefit from intensified platinum-based chemotherapy.

Maintenance of genomic integrity depends on homologous recombination, a conservative mechanism for error-free repair of DNA double-strand breaks (DSBs). In the absence of homologous recombination, error-prone DSB repair mechanisms such as nonhomologous end joining are invoked, leading to genomic instability [4-6]. This instability is thought to predispose to familial breast cancer in patients carrying germline mutations in *BRCA1* or *BRCA2,* genes involved in homologous recombination. Absence of homologous recombination offers a potential drug target for therapies that lead to DSBs during the DNA replication phase, when homologous recombination is the dominant DSB repair mechanism. Examples of these therapies are bifunctional alkylating agents and platinum compounds, which cause DNA crosslinks leading to DSBs during DNA replication, and poly(ADP-ribose)polymerase (PARP)-inhibitors [7,8], which inhibit repair of single-strand DNA breaks also resulting in DSBs during replication. Recent evidence indeed shows that *BRCA1/2*-mutated breast cancers are particularly sensitive to such agents [8-11]. This sensitivity is likely not restricted to *BRCA1/2*-mutated breast cancers, as it is thought that up to 30% of sporadic (germline *BRCA* wild-type) breast cancers have defects in homologous recombination repair, a phenotype that is often referred to as ‘BRCAness’ [12]. In order to identify sporadic breast cancers sensitive to DSB-inducing agents, many studies have focused on triple-negative (hormone receptor-negative and HER2-negative) breast cancers (TNBCs), as these cluster with BRCA1-mutated breast cancers within the basal-like molecular subtype [13,14]. Consequently, multiple trials with DSB-inducing agents have been performed in patients with TNBC and indeed have shown good responses to these agents, not only in *BRCA1-*mutation carriers [9,10]. However, in order to discern general chemosensitivity from agent-specific sensitivity, a randomized controlled trial (RCT) context is required, or, less ideal, a matched case-control setup [15].

We have previously employed array comparative genomic hybridization (aCGH) to assess the genomic profiles of *BRCA1*-mutated breast cancers [16]. It appeared that some sporadic breast cancers had aCGH patterns that resembled BRCA1-mutated breast cancers [17,18]. Furthermore, the BRCA1-like aCGH pattern was associated with benefit from a high-dose (HD) DSB-inducing regimen, and with a triple-negative (TN) phenotype [18].

*BRCA2*-mutated breast cancers show a similar distribution across the breast cancer subtypes as sporadic tumors (approximately 70% estrogen receptor (ER)- or progesterone receptor (PR)-positive) [19], and ways to select patients with sporadic ER-positive tumors sensitive to DSB-inducing agents have been lacking thus far.

Recently, a BRCA2-like CGH pattern was defined and found to be present in some sporadic breast cancers as well [17]. In contrast to the BRCA1-like CGH pattern, the BRCA2-like CGH pattern was frequently observed in ER-positive tumors.

Given the association of the BRCA1-like CGH pattern with benefit from HD DSB-inducing chemotherapy, we hypothesized that a positive BRCA-like^CGH^ status (the presence of a BRCA1-like and/or BRCA2-like CGH pattern) might identify, besides ER-negative, also ER-positive breast cancer patients who could benefit from DNA cross-linking agents. To explore this, we studied tumor specimens of breast cancer patients from a large RCT who had either received adjuvant, conventional 5-fluorouracil-epirubicin-cyclophosphamide (FE_90_C) chemotherapy followed by HD cyclophosphamide-thiotepa-carboplatin (CTC), a DNA cross-linking regimen, or conventional FE_90_C chemotherapy only [20]. We should note that this trial did not show superiority of HD-CTC over FEC, although subgroup analyses showed a better survival with HD-CTC in patients with HER2-negative tumors [21].

To enrich for cases likely to derive benefit from HD-CTC, we selected patients with HER2-negative tumors. By employing the BRCA-like^CGH^ status, we identified a subgroup of breast cancer patients with a remarkably good outcome after adjuvant HD-CTC compared to conventional FE_90_C chemotherapy, irrespective of hormone receptor status. Vice versa, we identified the subgroup that did not seem to derive any benefit from adjuvant HD-CTC.

## Methods

### Patients

Patients were part of a multicenter RCT performed in the Netherlands (1993 to 1999) [20]. In this trial, 885 breast cancer patients with at least four tumor-positive axillary lymph nodes but no distant metastases (stage III disease) had been randomized to conventional FE_90_C chemotherapy or the same therapy of which the last course had been replaced by HD-CTC chemotherapy with autologous stem cell support. Medical ethics committees of all participating centers approved the study protocol and all patients gave consent for participating in the study and publishing the study results. Trial centers were as follows (all in the Netherlands): The Netherlands Cancer Institute, Amsterdam; Free University Medical Center, Amsterdam; Academic Medical Center, Amsterdam; Leiden University Medical Center, Leiden; University Medical Center Groningen, Groningen; Maastricht University Medical Center, Maastricht; University Medical Center Nijmegen, Nijmegen; Erasmus Medical Center Cancer Institute, Rotterdam; Medisch Spectrum Twente, Enschede; University Medical Center Utrecht, Utrecht.

**Figure 1:**
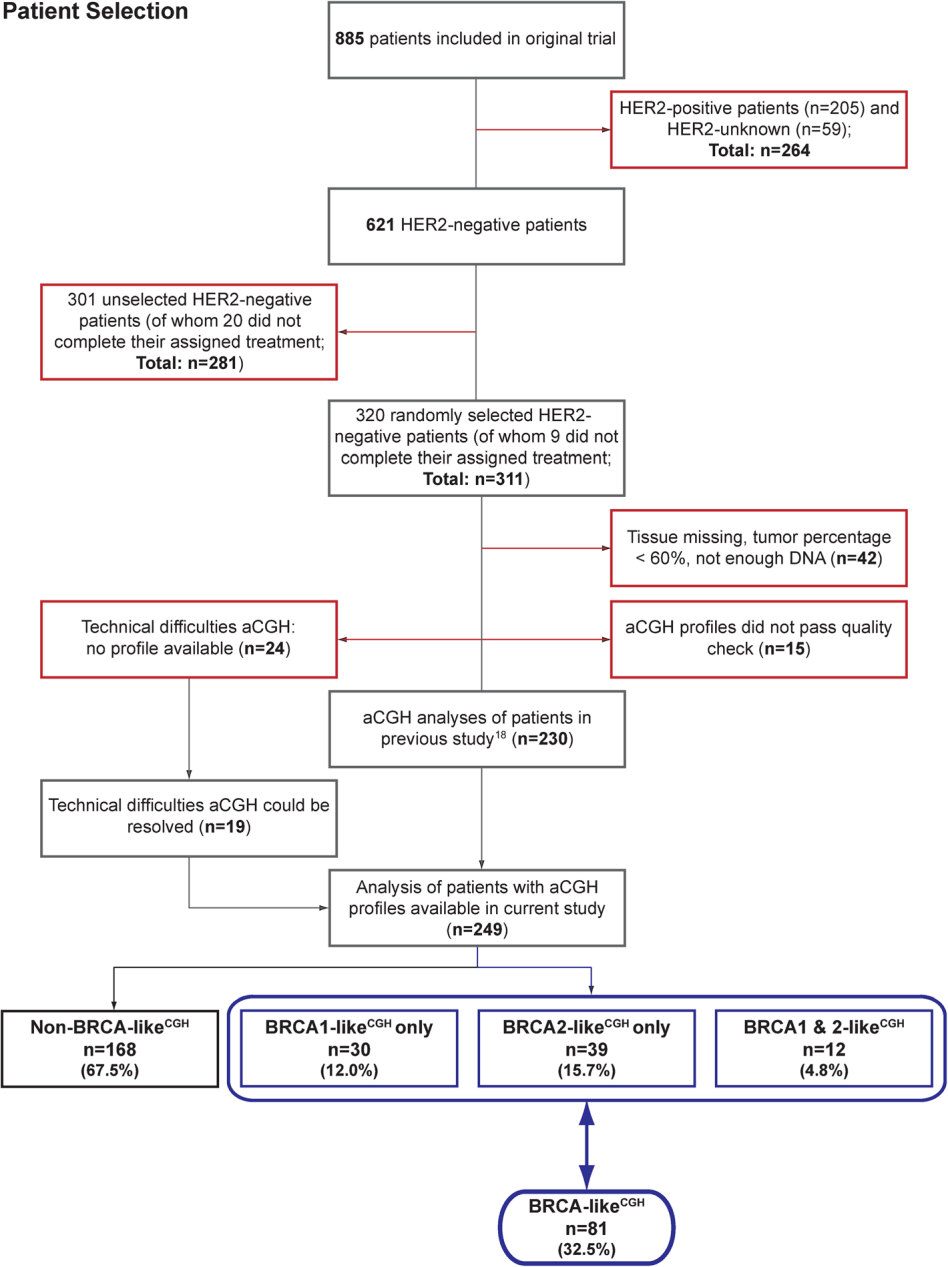
**Flow diagram of patient selection in the study.** Flow diagram depicting the number of patients and reasons for dropout (red boxes) and the number of patients remaining after each adjustment step (grey boxes). Tumors of 249 patients could be evaluated for the presence of the BRCA1-like^CGH^ and BRCA2-like^CGH^ pattern. The blue boxes at the bottom indicate the number of patients assigned to the BRCA1-like^CGH^, BRCA2-like^CGH^ and BRCA-like^CGH^ categories. aCGH, array comparative genomic hybridization.

For this study, we randomly selected a group of 320 (out of 621) HER2-negative breast cancer patients. This group was used previously to study the association of the BRCA1-like CGH pattern with benefit from HD-CTC [18]. Patients were included in the current study if formalin-fixed paraffin-embedded (FFPE) primary tumor tissue contained more than 60% tumor cells. Of these 320 patients we obtained aCGH profiles of 249 patients; the flow of the study and reasons for dropout are depicted in Figure 1. Patients selected for analyses did not differ in patient characteristics or treatment from those not selected for analyses (Table S7 in Additional file 1).

### Treatment

Conventional chemotherapy consisted of five courses of 5-fluorouracil 500 mg/m^2^, epirubicin 90 mg/m^2^, cyclophosphamide 500 mg/m^2^ (FE_90_C) given every three weeks [20]. The HD-CTC arm consisted of four FE_90_C courses, after which HD-CTC was administered (6,000 mg/m^2^ cyclophosphamide, 480 mg/m^2^ thiotepa and 1,600 mg/m^2^ carboplatin [20]. Patients in both treatment arms received radiotherapy and tamoxifen as described earlier [20].

### Array comparative genomic hybridization

aCGH patterns of 230 patients generated previously on a 3.5 k Human BAC array National Center for Biotechnical Information (NCBI)'s Gene Expression Omnibus platform number: GPL4560) platform were used in this study [18]. Tumors of 19 patients could additionally be analyzed (Figure 1). In short, genomic DNA was extracted from FFPE primary tumors [22]. For seven of these 19 additional patients, only lymph node tissue containing primary tumor tissue, removed at first diagnosis, was available. Three of these 19 samples had DNA concentrations too low for direct aCGH analysis and were amplified with the BioScore™ Screening and Amplification Kit (42440, Enzo Life Sciences, Farmingdale, NY, USA). Tumor and reference DNA was labeled according to the manufacturer’s instructions (Kreatech Biotechnology, Amsterdam, The Netherlands) and used for aCGH on the same 3.5 k Human BAC array platform, as previously described [23]. Quality of each aCGH pattern was determined using a profile quality and hybridization quality score, as published previously [18].

### BRCA-like^CGH^ status

Each aCGH profile was classified as either BRCA1-like or non-BRCA1-like and as either BRCA2-like or non-BRCA2-like as previously published based on respectively the evaluation of the BRCA1-like [18], and the BRCA2-like CGH pattern [17].

The BRCA-like^CGH^ class contained tumors with a BRCA1-like and/or a BRCA2-like CGH pattern; all other tumors were assigned to the non-BRCA-like^CGH^ class. The reproducibility of the BRCA-like^CGH^ status was tested by hybridizing 21 tumor DNA samples in duplicate. Two tumors in total switched classes from BRCA-like^CGH^ score upon second hybridization (in the analysis the first aCGH hybridization was used).

### Histopathology

Hematoxylin and eosin slides were scored for tumor percentages by a breast cancer pathologist (JW). ER, PR, P53, and HER2 status were determined by immunohistochemistry and scored as described previously [20,24].

### Statistical analyses

Groups of interest were tested for differences using Fisher’s exact tests and chi = square tests for trend. Recurrence-free survival (RFS) was defined as the time between randomization and appearance of local or regional recurrence, metastases or death from any cause, whichever came first [20]. Overall survival (OS) was calculated from randomization to death from any cause, or end of follow-up. Patients alive at last follow-up were censored at that time. Median RFS and OS were 7.7 and 8.3 years, respectively, for all 249 patients. Survival curves were computed using the Kaplan-Meier method and compared using log-rank tests; Cox regression was used to calculate hazard ratios (HR). To ensure a direct correlation between aCGH pattern and treatment received, only patients who completed their assigned treatment were analyzed (per protocol analysis).

This study was designed according to the predictive marker trial design ‘Indirect assessment: marker by treatment interaction design, test of interaction’ (design 2) [25]. With this design the hypothesis can be tested whether the treatment effect (that is HD-CTC versus FE_90_C) on survival in the presence of the marker (that is BRCA-like^CGH^) is significantly different from that in the absence of the marker (that is non-BRCA-like^CGH^) with a statistical test for interaction.

Evidence for non-proportional hazards was found; all multivariate Cox regression models were therefore stratified for number of lymph nodes (4-9 vs. ≥10) and TN status (ER <10% and PR <10% vs. other), which ensured hazards were proportional.

All calculations were performed using the statistical package SPSS 15.0 (for Windows) (SPSS Inc., Chicago, IL, USA). Figure S1 in Additional file 2 was generated using the ggplot2 package in R version 2.12.1.

## Results

### Frequency of BRCA-like^CGH^ status and patient characteristics

aCGH profiles could be obtained from 249 tumors. Thirty tumors were classified as BRCA1-like (BRCA1-like^CGH^), 39 tumors as BRCA2-like (BRCA2-like^CGH^) and 12 tumors as both BRCA1- and BRCA2-like^CGH^ (Figure 1); thereby assigning 81 patients to the BRCA-like^CGH^ class (81/249, 32%; Figure 1). Patients with BRCA-like^CGH^ tumors were generally younger and their tumors were more often ER-negative, PR-negative and poorly differentiated compared to patients with non-BRCA-like^CGH^ tumors (Table 1). BRCA1-like^CGH^ tumors were more often ER-negative (36/42, 86%; Table S1 in Additional file 1) than BRCA2-like^CGH^ tumors (16/51, 31%; Table S2 in Additional file 1). Figure S1 in Additional file 2 summarizes grade, receptor and BRCA-like^CGH^ status per patient.

**Table 1:** **Patient characteristics by BRCA-like**^**CGH **^**status**

**Variable**	**Patients with non-BRCA-like**^**CGH **^**tumors**		**Patients with BRCA-like**^**CGH **^**tumors**		***P *** **values**
	**n**	**%**	**n**	**%**	
**Total**	168	67.5	81	32.5	
**Treatment**					
FE_90_C chemotherapy	81	48.2	41	50.6	0.787
HD-CTC chemotherapy	87	51.8	40	49.4	
**Type of surgery**					
Breast-conserving therapy	33	19.6	18	22.2	0.620
Mastectomy	135	80.4	63	77.8	
**Age in categories**					
<40 years	34	20.2	27	33.3	0.032^*^
40 - 49 years	91	54.2	39	48.1	
≥50 years	43	25.6	15	18.5	
**Tumor classification**					
T1	32	19.0	15	18.5	0.642^*^
T2	112	66.7	51	63.0	
T3	23	13.7	14	17.3	
Unknown	1	0.6	1	1.2	
**Number of positive lymph nodes**					
4-9	109	64.9	54	66.7	0.887
≥10	59	35.1	27	33.3	
**Histologic grade**					
I	51	30.4	4	4.9	<0.001^*^
II	70	41.7	23	28.4	
III	42	25.0	50	61.7	
Not determined	5	3.0	4	4.9	
**Estrogen receptor status**					
Negative (<10%)	25	14.9	40	49.4	<0.001
Positive (≥10%)	143	85.1	41	50.6	
**Progesterone receptor status**					
Negative (<10%)	50	29.8	51	63.0	<0.001
Positive (≥10%)	118	70.2	28	34.6	
Unknown	0	0.0	2	2.5	
**Triple-negative status**					
Triple-negative	22	13.1	38	46.9	<0.001
ER or PR positive (>10%)	146	86.9	41	50.6	
Unknown	0	0.0	2	2.5	
**P53 status**					
<10%	99	58.9	43	53.1	0.087^*^
10 - 50%	48	28.6	11	13.6	
>50%	16	9.5	19	23.5	
Unknown	5	3.0	8	9.9	

*P* values: patients with unknown values were omitted. *P* values were calculated using the Fisher’s exact test, except for *chi-square test for trend. FE_90_C, 5-fluorouracil-epirubicin-cyclophosphamide; HD-CTC, high-dose cyclophosphamide-thiotepa-carboplatin; ER, estrogen receptor; PR, progesterone receptor.

Patient characteristics did not differ by treatment arm within the patient subgroups (with either a BRCA- or non-BRCA-like^CGH^ tumor; Table S3 in Additional file 1). In univariate Cox regression analyses, large pathological tumor size according to the tumor, node, metastasis (TNM) classification, high number of positive lymph nodes, poor Bloom-Richardson grade (BR grading system), TN status, aCGH BRCA1-like pattern and conventional FE_90_C treatment were significantly associated with decreased OS (Table S4 in Additional file 1). All further Cox regression analyses were therefore stratified for triple negativity and number of positive lymph nodes, and adjusted for pathological tumor size, BR grade, aCGH BRCA-like status and treatment.

Although aCGH BRCA1-like pattern was an adverse prognostic factor in univariate analysis (Table S4 in Additional file 1), it lost its prognostic value in multivariate analysis (Table S5 in Additional file 1), since it was highly associated with young age, TNBC, and poor histological grade (Table S1 in Additional file 1). The aCGH BRCA2-like pattern, however, was associated with an adverse prognosis in multivariate analysis (Table S5 in Additional file 1). One of the reasons that this was obscured in univariate analysis is that the BRCA1-like^CGH^ tumors ended up in the non- BRCA2-like^CGH^ tumor group, thereby confounding the analysis. This was corrected for in multivariate analysis (Table S5 in Additional file 1).

### Different treatment effects on survival between patients with BRCA-like^CGH^ and non-BRCA-like^CGH^ tumors

**Figure 2:**
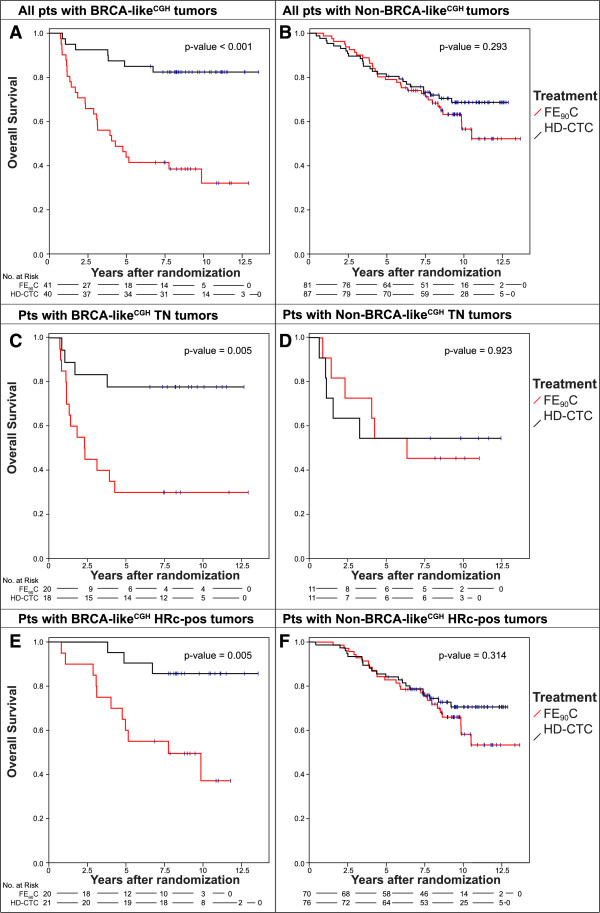
**Association of the BRCA-like**^**CGH **^**status with overall survival after HD-CTC and conventional chemotherapy.** Kaplan-Meier survival curves for OS were generated separately for all HER2-negative breast cancer patients with BRCA-like^CGH^**(A)** and with non-BRCA-like^CGH^**(B)** tumors; for the subgroup of TNBC patients with BRCA-like^CGH^**(C)** and with non-BRCA-like^CGH^**(D)** tumors; and for the subgroup of hormone receptor-positive, HER2-negative breast cancer patients with BRCA-like^CGH^**(E)** and with non-BRCA-like^CGH^ tumors **(F)**; who had been randomly assigned between HD-PB chemotherapy and conventional chemotherapy. FE_90_C, 5-fluorouracil, epirubicin, cyclophosphamide; HD-CTC, high-dose cyclophosphamide-thiotepa-carboplatin; HD-PB, high-dose platinum-based; HR-pos, hormone receptor-positive; OS, overall survival; TNBC, triple-negative breast cancer.

Patients with a BRCA-like^CGH^ tumor had a significantly better OS after HD-CTC compared with conventional FE_90_C (adjusted HR 0.19, 95% CI: 0.08 to 0.48, Table 2, Figure 2A), while there was no survival difference between treatment arms among patients with non-BRCA-like^CGH^ tumors (adjusted HR 0.90, 95% CI: 0.53 to 1.54, Table 2, Figure 2B). The effect of HD-CTC over conventional FE_90_C chemotherapy was significantly different between patients with BRCA-like^CGH^ tumors and non-BRCA-like^CGH^ tumors (test for interaction *P* = 0.004, Table 2). Similar results were obtained for RFS (Figure S2A and S2B in Additional file 2, test for interaction *P* = 0.003), and when BRCA1-like^CGH^ status and BRCA2-like^CGH^ status were analyzed separately (Table S5 in Additional file 1, Figure S3 in Additional file 1). The BRCA-like^CGH^ status retained its predictive capacity within the following subgroups: TNBC patients (Table 2, Figures 2C and 2D), ER-positive patients (Table 2, Figures 2E and 2F), patients younger than 45 years (Figure S4 in Additional file 1, Table S6 in Additional file 1), and showed a strong trend in patients with histological grade III tumors only (Figure S4 in Additional file 1, Table S6 in Additional file 1). We should note that subgroup analyses should be interpreted with caution due to small numbers in some subgroups.

**Table 2:** **Multivariate Cox proportional-hazard analysis of the risk of death (OS) and BRCA-like**^**CGH **^**status in all patients, patients with triple-negative tumors only and patients with hormone receptor-positive tumors only**

	**All patients**^**†**^				**Patients with TN tumors**^**‡**^				**Patients with HR-pos tumors**^**§**^			
**Variable**	**No. events/No. patients**	**Hazard ratio**	**95% CI**	***P *** **value**	**No. events/No. patients**	**Hazard ratio**	**95% CI**	***P *** **value**	**No. events/No. patients**	**Hazard ratio**	**95% CI**	***P *** **value**
**pT-stage**												
pT1/pT2	65/200	1.00			17/44	1.00			48/156	1.00		
pT3	22/37	1.93	1.16 - 3.21	0.012	10/13	2.46	1.03 - 5.88	0.043	12/24	1.71	0.88 - 3.32	0.114
**Histologic grade**												
I/II	48/147	1.00			7/15	1.00			41/132	1.00		
III	39/90	1.34	0.81 - 2.20	0.250	20/42	1.60	0.62 - 4.13	0.334	19/48	1.25	0.69 - 2.27	0.455
aCGH pattern*												
Non-BRCA-like^CGH^ tumor	56/162	1.00			10/21	1.00			46/141	1.00		
BRCA-like^CGH^ tumor	31/75	1.78	0.97 - 3.24	0.061	17/36	2.11	0.72 - 6.19	0.173	14/39	1.79	0.82 - 3.92	0.143
**BRCA-like**^**CGH **^tumor*												
FE_90_C chemotherapy	25/40	1.00			14/20	1.00	1.00		11/20	1.00		
HD-CTC chemotherapy	6/35	0.19^†^	0.08 - 0.48	<0.001	3/16	0.19^‡^	0.19^‡^	0.05 – 0.66	3/19	0.19^§^	0.05 - 0.71	0.013
**Non-BRCA-like**^**CGH **^tumor*			^†^Homogeneity: *P* = 0.004				^‡^Homogeneity: *P* = 0.034				^§^Homogeneity: *P* = 0.048	
FE_90_C chemotherapy	30/79	1.00			5/10	1.00			25/69	1.00		
HD-CTC chemotherapy	26/83	0.89^†^	0.52 - 1.50	5/11	5/11	1.31^‡^	0.37 - 4.64	0.676	21/72	0.82^§^	0.46 - 1.46	0.493

Three separate multivariate Cox regression models were run in all patients^†^, in patients with TNBC^‡^, and in patients with HR-positive tumors^§^ (see top row) and an *interaction term with treatment; the first model was stratified for number of lymph nodes (4-9 vs. ≥10) and triple-negative status (ER < 10% and PR < 10% vs. other) and based on 237 patients (12 patients contributing three events were excluded due to missing values for at least one of the variables shown). For patients with TN tumors and with HR-pos tumors only, models were stratified for lymph node status only. The TN subgroup analyses were based on 57 patients (three patients contributing two events were excluded due to missing values); for the HR-pos patients analyses were based on 180 patients (seven patients contributing zero events were excluded due to missing values). Test of homogeneity of both treatment-specific hazard ratios based on an interaction term: *P* = 0.004 (†), *P* = 0.034 (‡) and, *P* = 0.048 (§) OS, overall survival; TN, triple-negative; HR-pos, hormone receptor-positive; pT stage, pathological tumor size; aCGH, array comparative genomic hybridization; FE_90_C, 5-fluorouracil-epirubicin-cyclophosphamide; HD-CTC, high-dose cyclophosphamide-thiotepa-carboplatin.

### Effect of technical parameters on survival data

**Figure 3:**
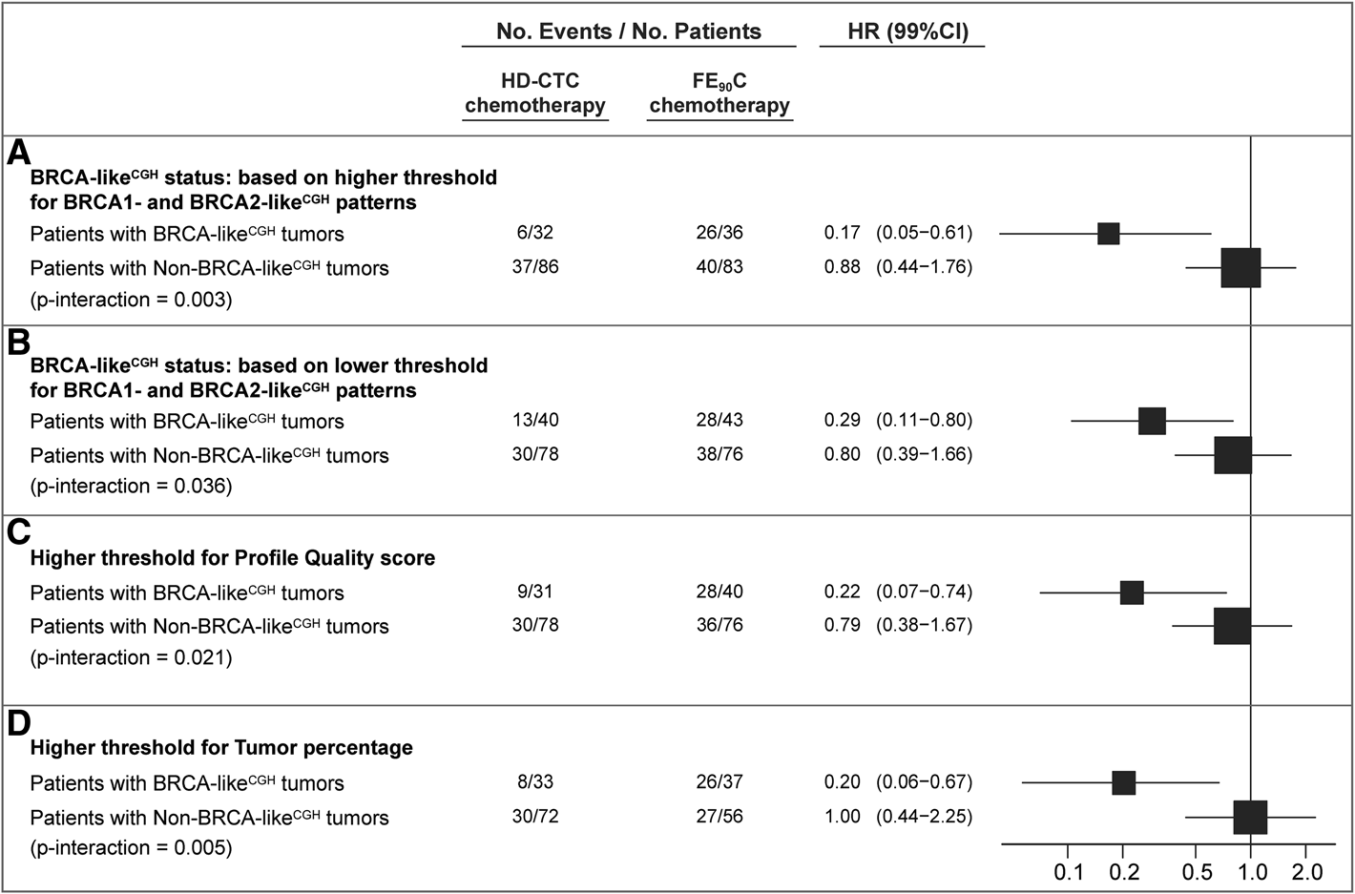
**Sensitivity analyses of the BRCA-like**^**CGH **^**status.** We varied the previously determined thresholds defining the BRCA-like^CGH^ status (that is the threshold of the BRCA1-like^CGH^ and BRCA2-like^CGH^ patterns (13, 14)) and the empirically chosen thresholds of the aCGH quality (profile-quality status) and the tumor percentage, and evaluated the influence on results for overall survival. **(A)** The thresholds of the BRCA1- and BRCA2-like^CGH^ patterns, which define the BRCA-like^CGH^ status, were increased by 0.1 (from 0.63 to 0.73 and from 0.5 to 0.6, respectively (13, 14)); **(B)** similarly, thresholds were decreased by 0.1; **(C)** The threshold determining aCGH quality was increased (from 0.85 to 0.95 (14)), resulting in a subgroup of 225 patients; **(D)** The threshold of tumor percentage was increased from 60% to 70% resulting in a subgroup of 198 patients. All analyses were stratified for number of lymph nodes (4-9 vs. ≥10) and double-negative ER/PR status (ER <10% and PR <10% vs. other) and adjusted for pathologic tumor size (T1 vs. T2 vs. T3), histologic grade (I vs. II vs. III) and BRCA-like^CGH^ status. aCGH, array comparative genomic hybridization; ER, estrogen receptor; FE_90_C, 5-fluorouracil, epirubicin, cyclophosphamide; HD-CTC, high-dose cyclophosphamide-thiotepa-carboplatin; PR progesterone receptor.

To determine the influence of the empirically chosen thresholds of the aCGH quality (profile-quality score), the minimal tumor percentage required for inclusion, and the influence of the previously determined thresholds defining the BRCA-like^CGH^ score (that is the threshold of the BRCA1-like^CGH^ and BRCA2-like^CGH^ patterns [17,18]), we varied the cutoffs around these thresholds and evaluated the influence of these changes on survival analyses. No substantial modification of the HRs of treatment among patients with BRCA-like^CGH^ or non-BRCA-like^CGH^ tumors (Figure 3), or of the tests for interaction (all *P* values remained significant, Figure 3), was observed.

## Discussion

In this study, we investigated whether we could identify a subgroup of HER2-negative patients who would derive substantial benefit from an intensified DNA DSB-inducing regimen, cyclophosphamide-thiotepa-carboplatin, with autologous stem cell support when compared to conventional FE_90_C [20]. We hypothesized that the aCGH patterns resembling *BRCA1*- or *2*-mutated breast cancers (BRCA-like^CGH^ status) would identify a subgroup of not only TN, but also ER-positive, HER2-negative breast cancer patients with tumors exquisitely sensitive to DNA DSB-inducing agents. Supporting evidence had come from a case report describing a patient with *BRCA2*-mutated metastatic breast cancer, who had been in continuous complete remission for 11 years after HD DNA-cross-linking chemotherapy with autologous stem cell support [26], from our own metastatic series [18], from two early PARP-inhibitor trials [8,11], and from preclinical work [7,27,28]. Indeed, in the current study breast cancer patients with a BRCA-like^CGH^ tumor had a markedly better OS after adjuvant HD-CTC than after conventional chemotherapy; this selective benefit was not present in patients with a non-BRCA-like^CGH^ tumor, as confirmed by a highly significant test for interaction. A substantial proportion of HER2-negative breast cancer patients had a BRCA-like^CGH^ tumor (81/249, 32%), which is close to the predicted proportion of BRCAness in sporadic breast cancers (30%) [12]. Interestingly, the proportion of ER-negative breast cancers among BRCA1-like^CGH^ and BRCA2-like^CGH^ breast cancers was similar to that reported for *BRCA1*-mutated and *BRCA2*-mutated breast cancers, respectively [12,19].

Fifty-one percent (41/81) of the BRCA-like^CGH^ tumors were ER-positive, making this the first study reporting on a potential marker for sensitivity to intensified DSB-inducing agents within the sporadic ER-positive breast cancer population.

Although there are indications that *BRCA2*-mutated breast cancers and BRCA2-like^CGH^ tumors are slightly more sensitive to standard chemotherapy than breast cancers not having these features [17,29,30], here we have shown that patients with BRCA2-like^CGH^ tumors, just like patients with BRCA1-like^CGH^ tumors, derive substantial more benefit from intensified, DNA DSB-inducing agents (CTC) than from conventional FE_90_C. Unfortunately, we could not determine whether a lower, non-myeloablative dose of CTC would also have resulted in a similarly improved outcome. This is important, since high-dose myeloablative chemotherapy has been abandoned in breast cancer treatment [3,31,32], although a small survival advantage has been reported for HER2-negative breast cancer in a recent meta-analysis [33]. In addition, it could also be that patients with BRCA-like tumors benefitted particularly from platinum rather than from the high-dose therapy. We cannot differentiate between these options due to the design of the original RCT. Another limitation of this study is that patients were not treated by a taxane, which is nowadays the standard of care for high-risk breast cancer. Finally, the randomized trial only included high-risk patients, younger than 55 years, with at least four tumor-positive axillary lymph nodes (stage III disease). Further studies are in progress to determine if these results are also applicable to other stages of breast cancer (NCT01057069 and NCT01646034).

There is evidence of a dose-response relationship for cyclophosphamide within a subgroup of breast cancers [34]. In the neoadjuvant setting, cT3-4 breast cancer patients with a *TP53*-mutation had a higher likelihood of pathological complete remission (pCR) and an 80% six-years RFS after intensified cyclophosphamide-based chemotherapy, but only a 50% six-year RFS after FEC-docetaxel (FEC-D) [34,35]. The *TP53* mutations were determined using a yeast-based screen for functional *TP53* mutations [36]. Interestingly, these specific types of *TP53* mutations have been found in high frequencies in *BRCA1*-mutated breast cancers [37] and could possibly function as an alternative marker for sensitivity to DNA cross-linking agents. In line with this reasoning, protein-truncating nonsense or frameshift *TP53* mutations were also found to predict good response (Miller-Payne score 3,4,5) to neoadjuvant cisplatin in cT2, TNBC patients [10]. At present the optimal intensified cyclophosphamide dose is unclear. In the B-25 trial breast cancer patients under 50 years of age with four to nine positive axillary lymph nodes benefitted significantly more from highly intensified (4*2,400 mg/m2 q 3 weeks) than from moderately intensified cyclophosphamide-based chemotherapy (4*1,200 mg/m2 q 3 weeks) [2]; similarly, in the CONSORT study (breast cancer patients with ≥4 axillary involved lymph nodes), intense dose-dense sequential epirubicin, paclitaxel and cyclophosphamide (3*2,500 mg/m2 q 2 weeks) significantly improved survival outcome compared with conventional chemotherapy [38]. Of course, in these two latter studies, no stratification based on a BRCAness marker was made, which might have resulted in a much more pronounced benefit in the BRCAness group.

The current study had been designed to test the hypothesis that breast cancers with HRD would derive substantially more benefit from intensified DNA DSB-inducing agents than tumors without HRD. We assumed that the BRCA-like^CGH^ status could be used as a proxy for breast cancers with HRD. Circumstantial evidence for this was derived from preclinical studies [4-8,12,39-43]. The presence of HRD is, however, not easy to establish in clinical samples since a gold standard for HRD does not exist. Therefore, we can only conclude that the BRCA-like^CGH^ status can be used to identify patients who derive substantial benefit from intensified DNA DSB-inducing agents. Whether the BRCA-like^CGH^ status can also be used to select patients who will derive substantial benefit from PARP-inhibitors is the subject of further studies.

As known, the performance of predictive biomarkers can only be studied in two comparable groups of patients where only one group has received the treatment of interest, thereby dissecting general chemosensitivity and/or prognosis from selective sensitivity to a particular treatment strategy [15,43,44]. The strength of the current study is that BRCA-like^CGH^ status has been tested in the context of a RCT with long-term follow-up. The BRCA-like^CGH^ status is probably not the only way to identify tumors that are sensitive to DNA DSB-inducing agents. Several potential predictive markers have been described in nonrandomized studies, such as RAD51 staining [45], gene expression profiling [46], telomere aberrations [47], *BRCA1*-promoter methylation, and *BRCA1* gene expression measurements [10].

To assess the play of chance, sensitivity analyses were performed showing that the results were robust with regard to the choice of several technical parameters. The association between HD-CTC benefit and the BRCA-like^CGH^ status remained significant with relatively few patients switching classes. Multivariate models, stratified for lymph node status and TNBC subtype, were run to assure that the observed differential treatment effects according to BRCA-like^CGH^ status were independent of histological grade and tumor size.

A technical restriction of this study was the use of an aCGH platform with a lower resolution than currently used platforms. The reason is that we had to employ the same platform for validation as we had used to build the classifiers. Of note, the low resolution regions employed in the BRCA-like^CGH^ classifier will, of course, not disappear when a higher resolution platform is employed. Clearly, it is not the platform, but rather the chromosomal regions that are important and our findings should be applicable on data generated by any technology that identifies DNA gains and losses. Finally, an advantage of the aCGH assay is that it requires limited amounts of DNA isolated from FFPE tissue.

In conclusion, we showed in a representative sample of 249 HER2-negative patients from a RCT that a BRCA-like^CGH^ classification was able to identify both ER-positive and TNBC patients who derived a marked benefit of intensified DNA cross-linking chemotherapy. Patients with BRCA-like^CGH^ tumors had about a five times lower risk of death after HD-CTC compared to FE_90_C chemotherapy, while no significant benefit was observed among patients with non-BRCA-like^CGH^ tumors. This finding strongly suggests the existence of breast cancer subtypes, defined by distinct CGH patterns that have a markedly improved outcome after treatment with an intensified DNA-cross-linking regimen and may explain why high-dose chemotherapy trials carried out in the general breast cancer population have remained negative in the past. This study should be considered as a biomarker study with an II-B level of evidence [15], since it tested the marker in a retrospective series. Therefore, before these results can be introduced into daily clinical practice, they should be validated in other controlled studies in which intensified alkylating regimens and/or PARP-inhibitors have been employed.

## Conclusions

aCGH patterns could differentiate between HER2-negative patients with a markedly improved outcome after adjuvant treatment with an intensified DNA DSB-inducing regimen (BRCA-like^CGH^ patients) and those without benefit (non-BRCA-like^CGH^ patients).

## Abbreviations

aCGH: array comparative genomic hybridization; BR: Bloom-Richardson grading system; CTC: cyclophosphamide-thiotepa-carboplatin; DSB: double-strand break; ER: estrogen receptor; FE_90_C: 5-fluorouracil-epirubicin-cyclophosphamide; FFPE: formalin-fixed paraffin-embedded; HD: high-dose; HRD: homologous recombination deficiency; OS: overall survival; PARP: poly(ADP-ribose)polymerase; pCR: pathological complete remission; PR: progesterone receptor; RCT: randomized controlled trial; RFS: recurrence-free survival; TN: triple negative; TNBC: triple negative breast cancer.

## Competing interests

S.C. Linn, M.A. Vollebergh and P.M. Nederlof are named inventors on a patent application for the aCGH BRCA1-like^CGH^ classifier and additionally with E.H. Lips as a contributor on a patent application for the aCGH BRCA2-like^CGH^ classifier used in this study. All other authors of this manuscript declare they have no financial and/or personal relationships with other people and/or organizations that could influence or bias their work.

## Authors’ contributions

SCL, LFAW, MH and SR were responsible for the study design. MAV and EHL coordinated the study. PMN, LFAW and JJ developed the research methods used. MAV, EHL, EGEdV and HvT took part in data collection. JW and MvdV performed all histopathological analyses. MAV and EHL carried out all experiments. MAV, EHL, MH and HvT performed the data analysis. MAV, EHL, SCL, PMN, LFAW, JJ, HvT, and MH took part in data interpretation. MAV, EHL, PMN, LFAW, JW, MvdV, EGEdV, HvT, JJ, MH, SR, SCL contributed to the revising or critical drafting of the manuscript. MAV, EHL, PMN, LFAW, JW, MvdV, EGEdV, HvT, JJ, MH, SR, SCL approved the final revised version of the manuscript. All authors, external and internal, had full access to all of the data (including statistical reports and tables) in the study.

## Supplementary Material

Additional file 1: Table S1 Patient characteristics by BRCA1-like^CGH^ status. **Table S2.** Patient characteristics by BRCA2-like^CGH^ status. **Table S3.** Patient characteristics by treatment arm and BRCA-like^CGH^ status. **Table S4.** Univariate Cox proportional hazard regression analysis of the risk of death (OS) after randomization. **Table S5.** Multivariate Cox proportional hazard analysis of the risk of death (OS) for patients with BRCA1-like^CGH^ tumors, or BRCA2-like^CGH^ tumors compared to patients with non-BRCA-like^CGH^ tumors. **Table S6.** Multivariate Cox proportional hazard analysis of the risk of death (OS) and BRCA-like^CGH^ status in patients with grade III tumors only and in patients younger than or equal to 45 years only **Table S7.** Distribution of clinicopathological variables between randomly selected HER2-negative patients included in this study and HER2-negative patients not in the current analysis (all completed their assigned treatment). Click here for file

Additional file 2: Figure S1 Overview of histological patient characteristics and aCGH classification per patient. **Figure S2.** Association of the BRCA-like CGH status with recurrence-free survival after HD-CTC and conventional FE_90_C chemotherapy. **Figure S3.** Association of the BRCA1-like CGH, the BRCA2-like CGH and the non-BRCA-like CGH status with overall survival after HD-CTC and conventional FE_90_C chemotherapy. **Figure S4.** Association of the BRCA-like CGH and the non-BRCA-like CGH status with overall survival after HD-CTC and conventional FE_90_C chemotherapy in patients with grade III tumors and in patients younger than 45 years. Click here for file
