# Supplementary material for: Genomic patterns resembling BRCA1- and BRCA2-mutated breast cancers predict benefit of intensified carboplatin-based chemotherapy
Source: Breast Cancer Res. 2014 May 15;16(3):R47. doi: 10.1186/bcr3655 (PMC4076636; doi:10.1186/bcr3655)
Supplement: Additional file 1: Table S1 — Patient characteristics by BRCA1-likeCGH status. Table S2. Patient characteristics by BRCA2-likeCGH status. Table S3. Patient characteristics by treatment arm and BRCA-likeCGH status. Table S4. Univariate Cox proportional hazard regression analysis of the risk of death (OS) after randomization. Table S5. Multivariate Cox proportional hazard analysis of the risk of death (OS) for patients with BRCA1-likeCGH tumors, or BRCA2-likeCGH tumors compared to patients with non-BRCA-likeCGH tumors. Table S6. Multivariate Cox proportional hazard analysis of the risk of death (OS) and BRCA-likeCGH status in patients with grade III tumors only and in patients younger than or equal to 45 years only Table S7. Distribution of clinicopathological variables between randomly selected HER2-negative patients included in this study and HER2-negative patients not in the current analysis (all completed their assigned treatment). [file bcr3655-S1.doc]

**Supplementary Table 1. Patient characteristics by BRCA1-like^CGH^ status**

| **Variable** |  | **Patients with**  **Non-BRCA1-like^CGH^ tumors** | |  | **Patients with**  **BRCA1-like^CGH^ tumors** | |  | **p values** |
| --- | --- | --- | --- | --- | --- | --- | --- | --- |
|  |  | n | % |  | n | % |  |  |
|  |  |  |  |  |  |  |  |  |
| **Total** |  | 207 | 83.1 |  | 42 | 16.9 |  |  |
|  |  |  |  |  |  |  |  |  |
| **Treatment** |  |  |  |  |  |  |  |  |
| FE_90_C chemotherapy | | 99 | 47.8 |  | 23 | 54.8 |  | 0.499 |
| HD-CTC Chemotherapy |  | 108 | 52.2 |  | 19 | 45.2 |  |
|  |  |  |  |  |  |  |  |  |
| **Type of surgery** |  |  |  |  |  |  |  |  |
| Breast conserving therapy |  | 37 | 17.9 |  | 14 | 33.3 |  | 0.035 |
| Mastectomy |  | 170 | 82.1 |  | 28 | 66.7 |  |
|  |  |  |  |  |  |  |  |  |
| **Age in categories** |  |  |  |  |  |  |  |  |
| < 40 years |  | 44 | 21.3 |  | 17 | 40.5 |  | 0.007* |
| 40 - 49 years |  | 110 | 53.1 |  | 20 | 47.6 |  |
| > 50 years |  | 53 | 25.6 |  | 5 | 11.9 |
|  |  |  |  |  |  |  |  |  |
| **Tumor classification** |  |  |  |  |  |  |  |  |
| T1 |  | 35 | 16.9 |  | 12 | 28.6 |  | 0.146* |
| T2 |  | 138 | 66.7 |  | 25 | 59.5 |
| T3 |  | 32 | 15.5 |  | 5 | 11.9 |
| Unknown |  | 2 | 1.0 |  | 0 | 0.0 |  |  |
|  |  |  |  |  |  |  |  |  |
| **No. of positive lymph nodes** | | |  |  |  |  |  |  |
| 4-9 |  | 136 | 65.7 |  | 27 | 64.3 |  | 0.860 |
| > 10 |  | 71 | 34.3 |  | 15 | 35.7 |
|  |  |  |  |  |  |  |  |  |
| **Histologic grade** |  |  |  |  |  |  |  |  |
| I |  | 55 | 26.6 |  | 0 | 0.0 |  | <0.001* |
| II |  | 88 | 42.5 |  | 5 | 11.9 |
| III |  | 58 | 28.0 |  | 34 | 81.0 |
| Not determined |  | 6 | 2.9 |  | 3 | 7.1 |  |  |
|  |  |  |  |  |  |  |  |  |
| **Estrogen receptor status** | |  |  |  |  |  |  |  |
| Negative (<10%) |  | 29 | 14.0 |  | 36 | 85.7 |  | <0.001* |
| Positive (>10%) |  | 178 | 86.0 |  | 6 | 14.3 |
|  |  |  |  |  |  |  |  |  |
| **Progesterone receptor status** | | |  |  |  |  |  |  |
| Negative (<10%) |  | 63 | 30.4 |  | 38 | 90.5 |  | <0.001* |
| Positive (>10%) |  | 144 | 69.6 |  | 2 | 4.8 |
| Unknown |  | 0 | 0.0 |  | 2 | 4.8 |  |  |
|  |  |  |  |  |  |  |  |  |
| **Triple-negative status** |  |  |  |  |  |  |  |  |
| Triple-negative | | 26 | 12.6 |  | 34 | 81.0 |  | <0.001* |
| ER or PR positive (> 10%) |  | 181 | 87.4 |  | 6 | 14.3 |  |
| Unknown |  | 0 | 0.0 |  | 2 | 4.8 |  |  |
|  |  |  |  |  |  |  |  |  |
| **P53 status** |  |  |  |  |  |  |  |  |
| < 10% |  | 127 | 61.4 |  | 15 | 35.7 |  | <0.001* |
| 10 – 50% |  | 54 | 26.1 |  | 5 | 11.9 |
| > 50% |  | 19 | 9.2 |  | 16 | 38.1 |  |
| Unknown |  | 7 | 3.4 |  | 6 | 14.3 |  |  |
|  |  |  |  |  |  |  |  |  |
| p values: patients with unknown values were omitted. p values were calculated using the Fisher’s exact test, except for ^*^Chi square test for trend. *Abbreviations:* FE_90_C, 5-fluorouracil-epirubicin-cyclophosphamide; HD-CTC, high-dose cyclophosphamide-thiotepa-carboplatin; ER, estrogen-receptor; PgR, progesterone-receptor. | | | | | | | | |

**Supplementary Table 2. Patient characteristics by BRCA2-like^CGH^ status**

| **Variable** |  | **Patients with**  **Non-BRCA2-like^CGH^** **tumors** | |  | **Patients with**  **BRCA2-like^CGH^ tumors** | |  | **p values** |
| --- | --- | --- | --- | --- | --- | --- | --- | --- |
|  |  | n | % |  | n | % |  |  |
|  |  |  |  |  |  |  |  |  |
| **Total** |  | 198 | 79.5 |  | 51 | 20.5 |  |  |
|  |  |  |  |  |  |  |  |  |
| **Treatment** |  |  |  |  |  |  |  |  |
| FE_90_C chemotherapy | | 97 | 49.0 |  | 25 | 49.0 |  | 1.000 |
| HD-CTC Chemotherapy |  | 101 | 51.0 |  | 26 | 51.0 |  |
|  |  |  |  |  |  |  |  |  |
| **Type of surgery** |  |  |  |  |  |  |  |  |
| Breast conserving therapy |  | 43 | 21.7 |  | 8 | 15.7 |  | 0.437 |
| Mastectomy |  | 155 | 78.3 |  | 43 | 84.3 |  |
|  |  |  |  |  |  |  |  |  |
| **Age in categories** |  |  |  |  |  |  |  |  |
| < 40 years |  | 43 | 21.7 |  | 18 | 35.3 |  | 0.173 |
| 40 - 49 years |  | 108 | 54.5 |  | 22 | 43.1 |  |
| > 50 years |  | 47 | 23.7 |  | 11 | 21.6 |
|  |  |  |  |  |  |  |  |  |
| **Tumor classification** |  |  |  |  |  |  |  |  |
| T1 |  | 39 | 19.7 |  | 8 | 15.7 |  | 0.281* |
| T2 |  | 131 | 66.2 |  | 32 | 62.7 |
| T3 |  | 27 | 13.6 |  | 10 | 19.6 |
| Unknown |  | 1 | 0.5 |  | 1 | 2.0 |  |  |
|  |  |  |  |  |  |  |  |  |
| **No. of positive lymph nodes** | | |  |  |  |  |  |  |
| 4-9 |  | 129 | 65.2 |  | 34 | 66.7 |  | 0.871 |
| > 10 |  | 69 | 34.8 |  | 17 | 33.3 |
|  |  |  |  |  |  |  |  |  |
| **Histologic grade** |  |  |  |  |  |  |  |  |
| I |  | 51 | 25.8 |  | 4 | 7.8 |  | 0.006* |
| II |  | 73 | 36.9 |  | 20 | 39.2 |
| III |  | 67 | 33.8 |  | 25 | 49.0 |
| Not determined |  | 7 | 3.5 |  | 2 | 3.9 |  |  |
|  |  |  |  |  |  |  |  |  |
| **Estrogen receptor status** | |  |  |  |  |  |  |  |
| Negative (<10%) |  | 49 | 24.7 |  | 16 | 31.4 |  | 0.372 |
| Positive (>10%) |  | 149 | 75.3 |  | 35 | 68.6 |
|  |  |  |  |  |  |  |  |  |
| **Progesterone receptor status** | | |  |  |  |  |  |  |
| Negative (<10%) |  | 76 | 38.4 |  | 25 | 49.0 |  | 0.203 |
| Positive (>10%) |  | 120 | 60.6 |  | 26 | 51.0 |
| Unknown |  | 2 | 1.0 |  | 0 | 0.0 |  |  |
|  |  |  |  |  |  |  |  |  |
| **Triple-negative status** |  |  |  |  |  |  |  |  |
| Triple-negative | | 44 | 22.2 |  | 16 | 31.4 |  | 0.202 |
| ER or PR positive (> 10%) |  | 152 | 76.8 |  | 35 | 68.6 |  |
| Unknown |  | 2 | 1.0 |  | 0 | 0.0 |  |  |
|  |  |  |  |  |  |  |  |  |
| **P53 status** |  |  |  |  |  |  |  |  |
| < 10% |  | 109 | 55.1 |  | 33 | 64.7 |  | 0.269* |
| 10 – 50% |  | 53 | 26.8 |  | 6 | 11.8 |
| > 50% |  | 28 | 14.1 |  | 7 | 13.7 |  |
| Unknown |  | 8 | 4.0 |  | 5 | 9.8 |  |  |
|  |  |  |  |  |  |  |  |  |
| p values: patients with unknown values were omitted. p values were calculated using the Fisher’s exact test, except for ^*^Chi square test for trend. *Abbreviations:* FE_90_C, 5-fluorouracil-epirubicin-cyclophosphamide; HD-CTC, high-dose cyclophosphamide-thiotepa-carboplatin; ER, estrogen-receptor; PgR, progesterone-receptor. | | | | | | | | |

**Supplementary Table 3. Patient characteristics by treatment arm and BRCA-like^CGH^ status**

| **Variable** |  | **Patients with**  **Non-BRCA-like**^CGH^ **tumors** | | | | |  | **Patients with**  **BRCA-like**^CGH^ **tumors** | | | | |
| --- | --- | --- | --- | --- | --- | --- | --- | --- | --- | --- | --- | --- |
|  |  | **FE_90_C Chemotherapy** | | **HD-CTC chemotherapy** | | **p values** |  | **FE_90_C Chemotherapy** | | **HD-CTC chemotherapy** | | **p values** |
|  |  | n | % | n | % |  |  | n | % | n | % |  |
|  |  |  |  |  |  |  |  |  |  |  |  |  |
| **Total** |  | 81 | 48.2 | 87 | 51.8 |  |  | 41 | 50.6 | 40 | 49.4 |  |
|  |  |  |  |  |  |  |  |  |  |  |  |  |
| **Age in categories** |  |  |  |  |  |  |  |  |  |  |  |  |
| < 40 years |  | 13 | 16.0 | 21 | 24.1 | 0.910* |  | 15 | 36.6 | 12 | 30.0 | 0.876* |
| 40 - 49 years |  | 50 | 61.7 | 41 | 47.1 |  |  | 18 | 43.9 | 21 | 52.5 |  |
| > 50 years |  | 18 | 22.2 | 25 | 28.7 |  |  | 8 | 19.5 | 7 | 17.5 |  |
|  |  |  |  |  |  |  |  |  |  |  |  |  |
| **Type of surgery** |  |  |  |  |  |  |  |  |  |  |  |  |
| Breast conserving therapy | | 15 | 18.5 | 18 | 20.7 | 0.846 |  | 10 | 24.4 | 8 | 20.0 | 0.790 |
| Mastectomy |  | 66 | 81.5 | 69 | 79.3 |  |  | 31 | 75.6 | 32 | 80.0 |  |
|  |  |  |  |  |  |  |  |  |  |  |  |  |
| **Tumor classification** | | |  |  |  |  |  |  |  |  |  |  |
| T1 |  | 14 | 17.3 | 18 | 20.7 | 0.787* |  | 8 | 19.5 | 7 | 17.5 | 0.587* |
| T2 |  | 56 | 69.1 | 56 | 64.4 |  |  | 24 | 58.5 | 27 | 67.5 |  |
| T3 |  | 11 | 13.6 | 12 | 13.8 |  |  | 9 | 22.0 | 5 | 12.5 |  |
| Unknown |  | 0 | 0.0 | 1 | 1.1 |  |  | 0 | 0.0 | 1 | 2.5 |  |
|  |  |  |  |  |  |  |  |  |  |  |  |  |
| **No. of positive lymph nodes** | | |  |  |  |  |  |  |  |  |  |  |
| 4-9 |  | 51 | 63.0 | 58 | 66.7 | 0.631 |  | 31 | 75.6 | 23 | 57.5 | 0.102 |
| > 10 |  | 30 | 37.0 | 29 | 33.3 |  |  | 10 | 24.4 | 17 | 42.5 |  |
|  |  |  |  |  |  |  |  |  |  |  |  |  |
| **Histologic grade** |  |  |  |  |  |  |  |  |  |  |  |  |
| I |  | 21 | 25.9 | 30 | 34.5 | 0.407* |  | 2 | 4.9 | 2 | 5.0 | 0.570* |
| II |  | 37 | 45.7 | 33 | 37.9 |  |  | 11 | 26.8 | 12 | 30.0 |  |
| III |  | 21 | 25.9 | 21 | 24.1 |  |  | 28 | 68.3 | 22 | 55.0 |  |
| Not determined |  | 2 | 2.5 | 3 | 3.4 |  |  | 0 | 0.0 | 4 | 10.0 |  |
|  |  |  |  |  |  |  |  |  |  |  |  |  |
| **Estrogen receptor status** | | |  |  |  |  |  |  |  |  |  |  |
| Negative (<10%) |  | 12 | 14.8 | 13 | 14.9 | 1.000 |  | 21 | 51.2 | 19 | 47.5 | 0.825 |
| Positive (>10%) |  | 69 | 85.2 | 74 | 85.1 |  |  | 20 | 48.8 | 21 | 52.5 |  |
|  |  |  |  |  |  |  |  |  |  |  |  |  |
| **Progesterone receptor status** | | |  |  |  |  |  |  |  |  |  |  |
| Negative (<10%) |  | 24 | 29.6 | 26 | 29.9 | 1.000 |  | 28 | 68.3 | 23 | 57.5 | 0.352 |
| Positive (>10%) |  | 57 | 70.4 | 61 | 70.1 |  |  | 12 | 29.3 | 16 | 40.0 |  |
| Unknown |  | 0 | 0.0 | 0 | 0.0 |  |  | 1 | 2.4 | 1 | 2.5 |  |
|  |  |  |  |  |  |  |  |  |  |  |  |  |
| **Triple-negative status** |  |  |  |  |  |  |  |  |  |  |  |  |
| Triple-negative | | 11 | 13.6 | 11 | 12.6 | 0.823 |  | 20 | 48.8 | 18 | 45.0 | 1.000 |
| ER or PR positive (> 10%) | | 70 | 86.4 | 76 | 87.4 |  |  | 20 | 48.8 | 21 | 52.5 |  |
| Unknown |  | 0 | 0.0 | 0 | 0.0 |  |  | 1 | 2.4 | 1 | 2.5 |  |
|  |  |  |  |  |  |  |  |  |  |  |  |  |
| **P53 status** |  |  |  |  |  |  |  |  |  |  |  |  |
| < 10% |  | 41 | 50.6 | 58 | 66.7 | 0.079* |  | 19 | 46.3 | 24 | 60.0 | 0.282* |
| 10 – 50% |  | 28 | 34.6 | 20 | 23.0 |  |  | 7 | 17.1 | 4 | 10.0 |  |
| > 50% |  | 9 | 11.1 | 7 | 8.0 |  |  | 11 | 26.8 | 8 | 20.0 |  |
| Unknown |  | 3 | 3.7 | 2 | 2.3 |  |  | 4 | 9.8 | 4 | 10.0 |  |
|  |  |  |  |  |  |  |  |  |  |  |  |  |
| p values: patients with unknown values were omitted; p values were calculated using the Fisher’s exact test, except for *Chi square test for trend. *Abbreviations:* FE_90_C, 5-fluorouracil-epirubicin-cyclophosphamide; HD-CTC, high-dose cyclophosphamide-thiotepa-carboplatin; ER, estrogen-receptor; PgR, progesterone-receptor. | | | | | | | | | | | | |

**Supplementary Table 4. Univariate Cox proportional-hazard regression analysis of the risk of death (OS) after randomization**

| **Variable** |  | **No. Events / No. patients** |  | **Hazard Ratio** |  | **95% CI** |  | **p values** |
| --- | --- | --- | --- | --- | --- | --- | --- | --- |
|  |  |  |  |  |  |  |  |  |
| **Age** |  |  |  |  |  |  |  |  |
| Continuously |  | 90 / 249 |  | 0.99 |  | 0.96 – 1.02 |  | 0.545 |
|  |  |  |  |  |  |  |  |  |
| **Type of surgery** |  |  |  |  |  |  |  |  |
| Breast conserving therapy |  | 19 / 51 |  | 1.00 |  |  |  |  |
| Mastectomy |  | 71 / 198 |  | 0.94 |  | 0.57 – 1.57 |  | 0.821 |
|  |  |  |  |  |  |  |  |  |
| **Pathological tumor classification** | | |  |  |  |  |  |  |
| T1 |  | 16 / 47 |  | 1.00 |  |  |  |  |
| T2 |  | 52 / 163 |  | 0.91 |  | 0.52 – 1.59 |  | 0.734 |
| T3 |  | 22 / 37 |  | 2.27 |  | 1.19 – 4.32 |  | 0.013 |
|  |  |  |  |  |  |  |  |  |
| **No. of positive lymph nodes** |  |  |  |  |  |  |  |  |
| 4-9 |  | 50 / 163 |  | 1.00 |  |  |  |  |
| > 10 |  | 40 / 86 |  | 1.73 |  | 1.14 – 2.62 |  | 0.010 |
|  |  |  |  |  |  |  |  |  |
| **Histologic grade** |  |  |  |  |  |  |  |  |
| I |  | 15 / 55 |  | 1.00 |  |  |  |  |
| II |  | 33 / 93 |  | 1.40 |  | 0.76 – 2.58 |  | 0.280 |
| III |  | 40 / 92 |  | 1.98 |  | 1.09 – 3.58 |  | 0.024 |
|  |  |  |  |  |  |  |  |  |
| **Estrogen receptor status** |  |  |  |  |  |  |  |  |
| Negative (<10%) |  | 31 / 65 |  | 1.00 |  |  |  |  |
| Positive (>10%) |  | 59 / 184 |  | 0.48 |  | 0.31 – 0.75 |  | 0.001 |
|  |  |  |  |  |  |  |  |  |
| **Progesterone receptor status** |  |  |  |  |  |  |  |  |
| Negative (<10%) |  | 45 / 101 |  | 1.00 |  |  |  |  |
| Positive (>10%) |  | 44 / 146 |  | 0.56 |  | 0.37 – 0.85 |  | 0.006 |
|  |  |  |  |  |  |  |  |  |
| **Triple-negative status** |  |  |  |  |  |  |  |  |
| Triple-negative |  | 29 / 60 |  | 1.00 |  |  |  |  |
| ER or PR positive (> 10%) |  | 60 / 187 |  | 0.48 |  | 0.31 – 0.75 |  | 0.001 |
|  |  |  |  |  |  |  |  |  |
| **P53 status** |  |  |  |  |  |  |  |  |
| < 10% |  | 52 / 142 |  | 1.00 |  |  |  |  |
| 10 – 50% |  | 18 / 59 |  | 0.73 |  | 0.43 – 1.25 |  | 0.246 |
| > 50% |  | 14 / 35 |  | 1.15 |  | 0.64 – 2.08 |  | 0.640 |
|  |  |  |  |  |  |  |  |  |
| **Treatment** |  |  |  |  |  |  |  |  |
| FE_90_C Chemotherapy |  | 57 / 122 |  | 1.00 |  |  |  |  |
| HD-CTC Chemotherapy |  | 33 / 127 |  | 0.48 |  | 0.31 – 0.74 |  | 0.001 |
|  |  |  |  |  |  |  |  |  |
| **aCGH BRCA-like status** |  |  |  |  |  |  |  |  |
| Non-BRCA-like^CGH^ tumor |  | 57 / 168 |  | 1.00 |  |  |  |  |
| BRCA-like^CGH^ tumor |  | 33 / 81 |  | 1.40 |  | 0.91 – 2.15 |  | 0.127 |
|  |  |  |  |  |  |  |  |  |
| **aCGH BRCA1-pattern** |  |  |  |  |  |  |  |  |
| Non-BRCA1-like^CGH^ tumor |  | 71 / 207 |  | 1.00 |  |  |  |  |
| BRCA1-like^CGH^ tumor |  | 19 / 42 |  | 1.79 |  | 1.08 – 2.98 |  | 0.024 |
|  |  |  |  |  |  |  |  |  |
| **aCGH BRCA2-pattern** |  |  |  |  |  |  |  |  |
| Non-BRCA2-like^CGH^ tumor |  | 69 / 198 |  | 1.00 |  |  |  |  |
| BRCA2-like^CGH^ tumor |  | 21 / 51 |  | 1.28 |  | 0.79 – 2.09 |  | 0.319 |
|  |  |  |  |  |  |  |  |  |
| Number of events is not equal for all variables, since some patients have missing data; maximum missing variables (i.e. events) is 6 /112. *Abbreviations:* CI, confidence interval; ER, estrogen-receptor; PgR, progesterone-receptor; FE_90_C, 5-fluorouracil-epirubicin-cyclophosphamide; HD-CTC, high-dose cyclophosphamide-thiotepa-carboplatin; CGH, comparative genomic hybridization; HD-CTC, high-dose. | | | | | | | | |

**Supplementary Table 5. Multivariate Cox proportional-hazard analysis of the risk of death (OS) for patients with BRCA1-like^CGH^ tumors, or BRCA2-like^CGH^ tumors compared to patients with non-BRCA-like^CGH^ tumors**

|  |  | **BRCA1-like^CGH^ pattern*** | | | | | |  | **BRCA2-like^CGH^ pattern*** | | | |
| --- | --- | --- | --- | --- | --- | --- | --- | --- | --- | --- | --- | --- |
| **Variable** |  | **No. Events /**  **No. patients** | **Hazard Ratio** | **95% CI** | | **p value** | |  | **No. Events /**  **No. patients** | **Hazard Ratio** | **95% CI** | **p value** |
|  |  |  |  |  | |  | |  |  |  |  |  |
| **p T-stage** |  |  |  |  | |  | |  |  |  |  |  |
| pT1 / pT2 |  | 54 / 172 | 1.00 |  | |  | |  | 58 / 177 | 1.00 |  |  |
| pT3 |  | 19 / 28 | 2.55 | 1.44 – 4.50 | | 0.001 | |  | 18 / 33 | 1.75 | 1.01 – 3.04 | 0.047 |
| 0.047 |  |  |  |  | |  | |  |  |  |  |  |
| **Histologic grade** |  |  |  |  | |  | |  |  |  |  |  |
| I / II |  | 39 / 126 | 1.00 |  | |  | |  | 47 / 144 | 1.00 |  |  |
| III |  | 34 / 74 | 1.83 | 1.05 – 3.18 | | 0.033 | |  | 29 / 66 | 1.21 | 0.71 – 2.07 | 0.487 |
|  |  |  |  |  | |  | |  |  |  |  |  |
| **aCGH pattern*** |  |  |  |  | | |  |  |  |  |  |  |
| Non-BRCA-like^CGH^ tumor |  | 56 / 162 | 1.00 |  |  | | |  | 56 / 162 | 1.00 |  |  |
| BRCA1-like^CGH^ or BRCA2-like^CGH^ tumor |  | 17 / 38 | 1.68 | 0.77 – 3.66 | 0.194 | | |  | 20 / 48 | 1.99 | 1.02 – 3.88 | 0.043 |
|  |  |  |  |  |  | | |  |  |  |  |  |
| **BRCA1- like^CGH^ or BRCA2-like^CGH^ tumor**^†,‡^ | | |  |  |  | | |  |  |  |  |  |
| FE_90_C chemotherapy |  | 14 / 22 | 1.00 |  |  | | |  | 16 / 25 | 1.00 |  |  |
| HD-CTC chemotherapy |  | 3 / 16 | 0.21^†^ | 0.06 – 0.73 | 0.015 | | |  | 4 / 23 | 0.18^‡^ | 0.06 – 0.56 | 0.003 |
|  |  |  |  | ^†^ Homogeneity: p=0.035 | | | |  |  |  | ^‡^ Homogeneity: p=0.014 | |
| **Non-BRCA-like^CGH^ tumor**^†,‡^ |  |  |  |  |  |  |  |  |  |  |  |  |
| FE_90_C chemotherapy |  | 30 / 79 | 1.00 |  |  |  |  |  | 30 / 79 | 1.00 |  |  |
| HD-CTC chemotherapy |  | 26 / 83 | 0.91^†^ | 0.53 – 1.54 | 0.715 | | |  | 26 / 83 | 0.86^‡^ | 0.51 – 1.47 | 0.589 |
|  |  |  |  |  |  | | |  |  |  |  |  |
|  |  |  |  |  |  | | |  |  |  |  |  |
| *Two separate multivariate Cox regression models were run with either the BRCA1-like^CGH^ pattern or the BRCA2-like^CGH^ pattern; and an interaction term with treatment; all models were stratified for number of lymph nodes (4-9 vs. >10) and triple-negative status (ER<10% and PR<10% vs. other) and based on 200 patients (10 patients contributing 3 events were excluded due to missing values for at least one of the variables shown) in the analyses with BRCA1-like^CGH^ status; and on 210 patients (9 patients contributing 2 events were excluded) in the analyses with BRCA2-like^CGH^ status. Test of homogeneity of both treatment-specific hazard ratios based on an interaction term: p=0.035 (†) and p= 0.014 (‡). *Abbreviations:* p T-stage, pathological Tumor size; aCGH, array Comparative Genomic Hybridization; FE_90_C, 5-fluorouracil-epirubicin-cyclophosphamide; HD-CTC, high-dose cyclophosphamide-thiotepa-carboplatin. | | | | | | | | | | | | |

**Supplementary Table 6. Multivariate Cox proportional-hazard analysis of the risk of death (OS) and BRCA-like^CGH^ status status in patients with grade-III tumors only and in patients younger than or equal to 45 years only**

|  |  | **Patients with grade-III tumors only*** | | | |  | **Patients < 45 years*** | | | |
| --- | --- | --- | --- | --- | --- | --- | --- | --- | --- | --- |
| **Variable** |  | **No. Events /**  **No. patients** | **Hazard Ratio** | **95% CI** | **p value** |  | **No. Events /**  **No. patients** | **Hazard Ratio** | **95% CI** | **p value** |
|  |  |  |  |  |  |  |  |  |  |  |
| **p T-stage** |  |  |  |  |  |  |  |  |  |  |
| pT1 / pT2 |  | 31 / 79 | 1.00 |  |  |  | 28 / 97 | 1.00 |  |  |
| pT3 |  | 8 / 11 | 1.85 | 0.79 – 4.32 | 0.158 |  | 10 / 15 | 2.53 | 1.13 – 5.67 | 0.024 |
| 0.047 |  |  |  |  |  |  |  |  |  |  |
| **Histologic grade** |  |  |  |  |  |  |  |  |  |  |
| I / II |  |  | NA |  |  |  | 21 / 67 | 1.00 |  |  |
| III |  |  | NA |  |  |  | 17 / 45 | 1.44 | 0.67 – 3.11 | 0.351 |
|  |  |  |  |  |  |  |  |  |  |  |
| **aCGH pattern*** |  |  |  |  |  |  |  |  |  |  |
| Non-BRCA-like^CGH^ tumor |  | 20 / 41 | 1.00 |  |  |  | 21 / 71 | 1.00 |  |  |
| BRCA-Like^CGH^ tumor |  | 19 / 49 | 1.07 | 0.47 – 2.41 | 0.879 |  | 17 / 41 | 2.88 | 1.19 – 6.96 | 0.019 |
|  |  |  |  |  |  |  |  |  |  |  |
| **BRCA-like^CGH^ tumor**^†,‡^ |  |  |  |  |  |  |  |  |  |  |
| FE_90_C chemotherapy |  | 15 / 27 | 1.00 |  |  |  | 16 / 23 | 1.00 |  |  |
| HD-CTC chemotherapy |  | 4 / 22 | 0.25^†^ | 0.08 – 0.78 | 0.016 |  | 1 / 18 | 0.06^‡^ | 0.008 – 0.46 | 0.007 |
|  |  |  |  | ^†^Homogeneity: p=0.080 | |  |  |  | ^‡^ Homogeneity: p=0.024 | |
| **Non-BRCA-like^CGH^ tumor**^†,‡^ |  |  |  |  |  |  |  |  |  |  |
| FE_90_C chemotherapy |  | 11 / 21 | 1.00 |  |  |  | 12 / 34 | 1.00 |  |  |
| HD-CTC chemotherapy |  | 9 / 20 | 0.92^†^ | 0.38 – 2.26 | 0.855 |  | 9 / 37 | 0.77^‡^ | 0.32 – 1.84 | 0.550 |
|  |  |  |  |  |  |  |  |  |  |  |
|  |  |  |  |  |  |  |  |  |  |  |
| *Two separate multivariate Cox regression models were run in above-mentioned subgroups; and an interaction term with treatment; all models were stratified for number of lymph nodes (4-9 vs. >10) and triple-negative status (ER<10% and PR<10% vs. other) and based on 90 patients (2 patients contributing 1 event were excluded due to missing values for at least one of the variables shown) in the analyses with patients with grade-III tumors only; and on 112 patients (4 patients contributing 2 events were excluded due to missing values) in the analyses of patients < 45 years. Test of homogeneity of both treatment-specific hazard ratios based on an interaction term: p=0.080(^†^) and p= 0.024 (‡). *Abbreviations:* p T-stage, pathological Tumor size; NA, not applicable; aCGH, array Comparative Genomic Hybridization; FE_90_C, 5-fluorouracil-epirubicin-cyclophosphamide; HD-CTC, high-dose cyclophosphamide-thiotepa-carboplatin. | | | | | | | | | | |

**Supplementary Table 7. Distribution of clinicopathological variables between randomly selected HER2-negative patients included in this study and HER2-negative patients not in the current analysis (all completed their assigned treatment).**

| **Variable** |  | **Total** | |  | **In analysis with aCGH classifier** | |  | **Not in current analysis** | |  | **p values** |
| --- | --- | --- | --- | --- | --- | --- | --- | --- | --- | --- | --- |
|  |  | **n** | **(%)** |  | **n** | **(%)** |  | **n** | **(%)** |  |  |
|  |  |  |  |  |  |  |  |  |  |  |  |
| **Total** |  | **592** | **100.0** |  | **249** | **42.1** |  | **343** | **57.9** |  |  |
|  |  |  |  |  |  |  |  |  |  |  |  |
| **Treatment** |  |  |  |  |  |  |  |  |  |  |  |
| FE_90_C chemotherapy |  | 298 | 50.3 |  | 122 | 49.0 |  | 176 | 51.3 |  | 0.618 |
| HD-CTC chemotherapy |  | 294 | 49.7 |  | 127 | 51.0 |  | 167 | 48.7 |  |
|  |  |  |  |  |  |  |  |  |  |  |  |
| **Age in categories** |  |  |  |  |  |  |  |  |  |  |  |
| < 40 years |  | 133 | 22.5 |  | 61 | 24.5 |  | 72 | 21.0 |  | 0.665* |
| 40 - 49 years |  | 324 | 54.7 |  | 130 | 52.2 |  | 194 | 56.6 |  |
| > 50 years |  | 135 | 22.8 |  | 58 | 23.3 |  | 77 | 22.4 |  |
|  |  |  |  |  |  |  |  |  |  |  |  |
| **Type of surgery** |  |  |  |  |  |  |  |  |  |  |  |
| Breast conserving therapy |  | 135 | 22.8 |  | 51 | 20.5 |  | 84 | 24.5 |  | 0.276 |
| Mastectomy |  | 457 | 77.2 |  | 198 | 79.5 |  | 259 | 75.5 |  |
|  |  |  |  |  |  |  |  |  |  |  |  |
| **Tumor classification** |  |  |  |  |  |  |  |  |  |  |  |
| T1 |  | 136 | 23.0 |  | 47 | 18.9 |  | 89 | 25.9 |  | 0.222^*^ |
| T2 |  | 357 | 60.3 |  | 163 | 65.5 |  | 194 | 56.6 |  |
| T3 |  | 90 | 15.2 |  | 37 | 14.9 |  | 53 | 15.5 |  |
| Unknown |  | 9 | 1.5 |  | 2 | 0.8 |  | 7 | 2.0 |  |  |
|  |  |  |  |  |  |  |  |  |  |  |  |
| **No. of positive lymph nodes** |  |  |  |  |  |  |  |  |  |  |  |
| 4-9 |  | 384 | 64.9 |  | 163 | 65.5 |  | 221 | 64.4 |  | 0.862 |
| > 10 |  | 208 | 35.1 |  | 86 | 34.5 |  | 122 | 35.6 |  |
|  |  |  |  |  |  |  |  |  |  |  |  |
| **Histologic grade** |  |  |  |  |  |  |  |  |  |  |  |
| I |  | 137 | 23.1 |  | 55 | 22.1 |  | 82 | 23.9 |  | 0.702^*^ |
| II |  | 221 | 37.3 |  | 93 | 37.3 |  | 128 | 37.3 |  |
| III |  | 217 | 36.7 |  | 92 | 36.9 |  | 125 | 36.4 |  |
| Not determined |  | 17 | 2.9 |  | 9 | 3.6 |  | 8 | 2.3 |  |  |
|  |  |  |  |  |  |  |  |  |  |  |  |
| **Estrogen receptor status** |  |  |  |  |  |  |  |  |  |  |  |
| Negative (<10%) |  | 140 | 23.6 |  | 65 | 26.1 |  | 75 | 21.9 |  | 0.242 |
| Positive (>10%) |  | 451 | 76.2 |  | 184 | 73.9 |  | 267 | 77.8 |  |
| Unknown |  | 1 | 0.2 |  | 0 | 0.0 |  | 1 | 0.3 |  |  |
|  |  |  |  |  |  |  |  |  |  |  |  |
| **Progesterone receptor status** | | |  |  |  |  |  |  |  |  |  |
| Negative (<10%) |  | 213 | 36.0 |  | 101 | 40.6 |  | 112 | 32.7 |  | 0.081 |
| Positive (>10%) |  | 368 | 62.2 |  | 146 | 58.6 |  | 222 | 64.7 |  |
| Unknown |  | 11 | 1.9 |  | 2 | 0.8 |  | 9 | 2.6 |  |  |
|  |  |  |  |  |  |  |  |  |  |  |  |
| **P53 status** |  |  |  |  |  |  |  |  |  |  |  |
| < 10% |  | 329 | 55.6 |  | 142 | 57.0 |  | 187 | 54.5 |  | 0.534* |
| 10 – 50% |  | 134 | 22.6 |  | 59 | 23.7 |  | 75 | 21.9 |  |
| > 50% |  | 91 | 15.4 |  | 35 | 14.1 |  | 56 | 16.3 |  |
| Unknown |  | 38 | 6.4 |  | 13 | 5.2 |  | 25 | 7.3 |  |  |
|  |  |  |  |  |  |  |  |  |  |  |  |
|  |  |  |  |  |  |  |  |  |  |  |  |
| p values: patients with unknown values were omitted; p values were calculated using the Fisher exact, except for ^*^Chi square test for trend. *Abbreviations:* aCGH, array Comparative Genomic Hybridization; FE_90_C, 5-fluorouracil-epirubicin-cyclophosphamide; HD-CTC, high-dose cyclophosphamide-thiotepa-carboplatin. | | | | | | | | | | | |
