# Supplementary figures and images for: Genomic patterns resembling BRCA1- and BRCA2-mutated breast cancers predict benefit of intensified carboplatin-based chemotherapy
Source: Breast Cancer Res. 2014 May 15;16(3):R47. doi: 10.1186/bcr3655 (PMC4076636; doi:10.1186/bcr3655)

Supplementary Figure 1

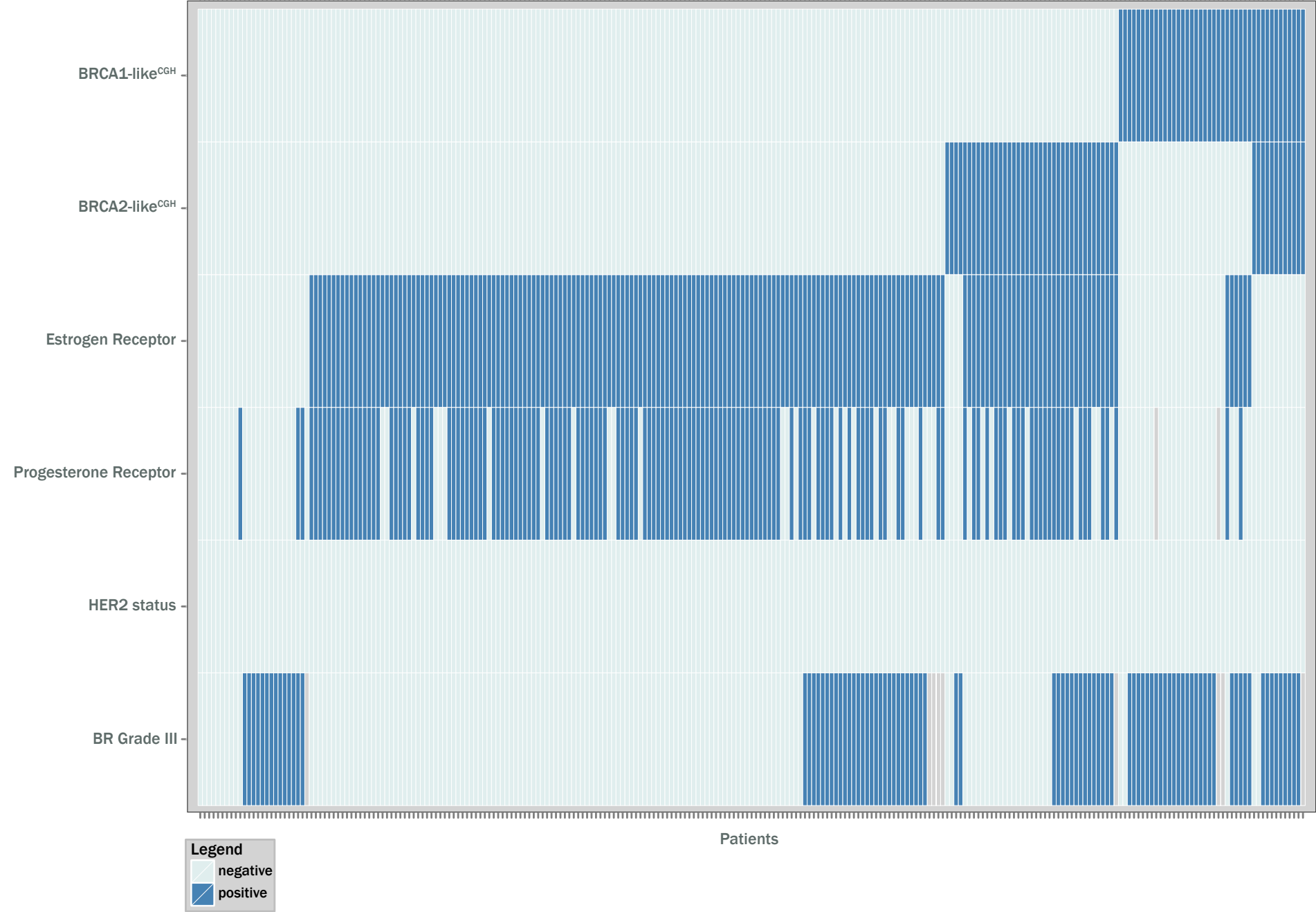

Supplementary Figure 2

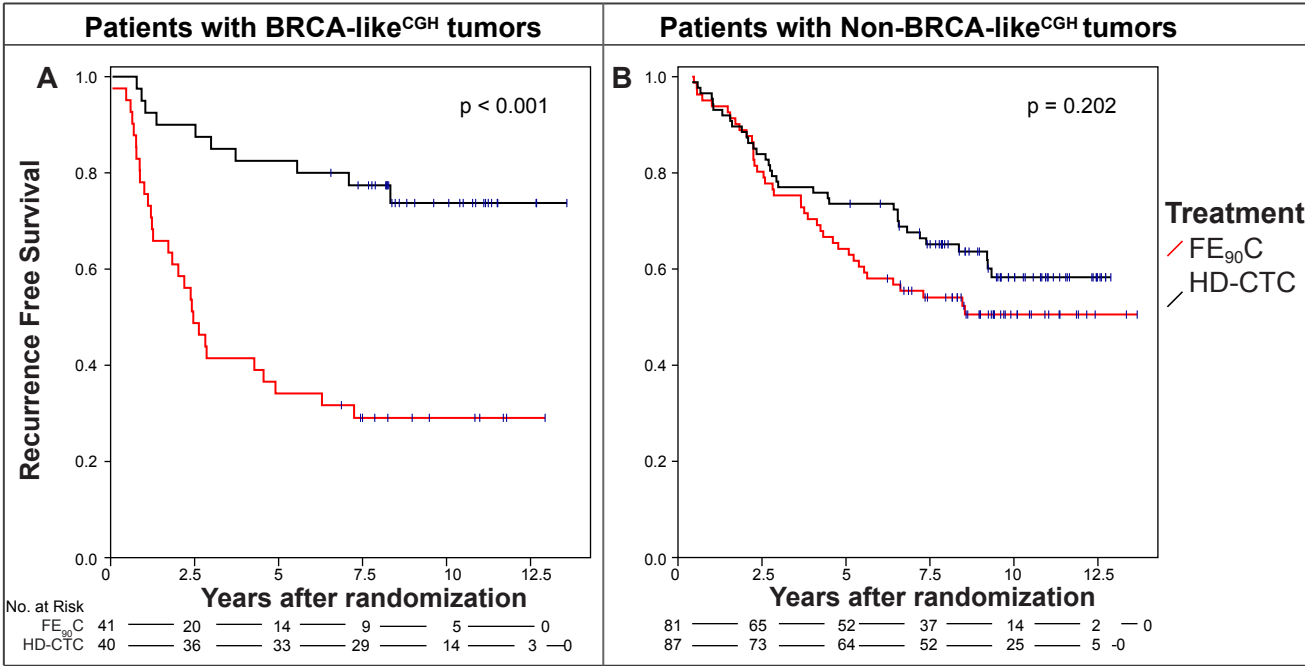

Supplementary Figure 3

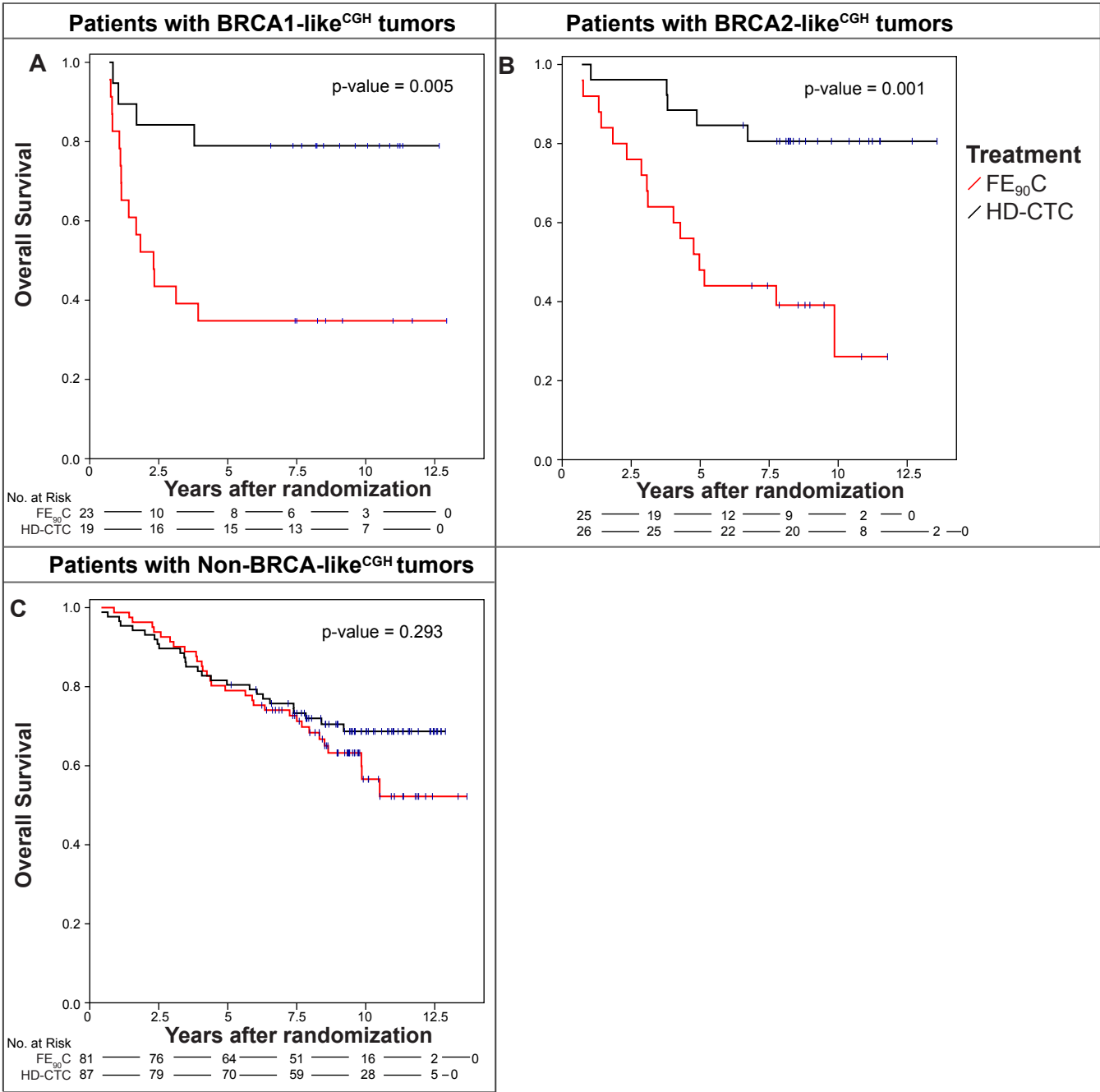

Supplementary Figure 4

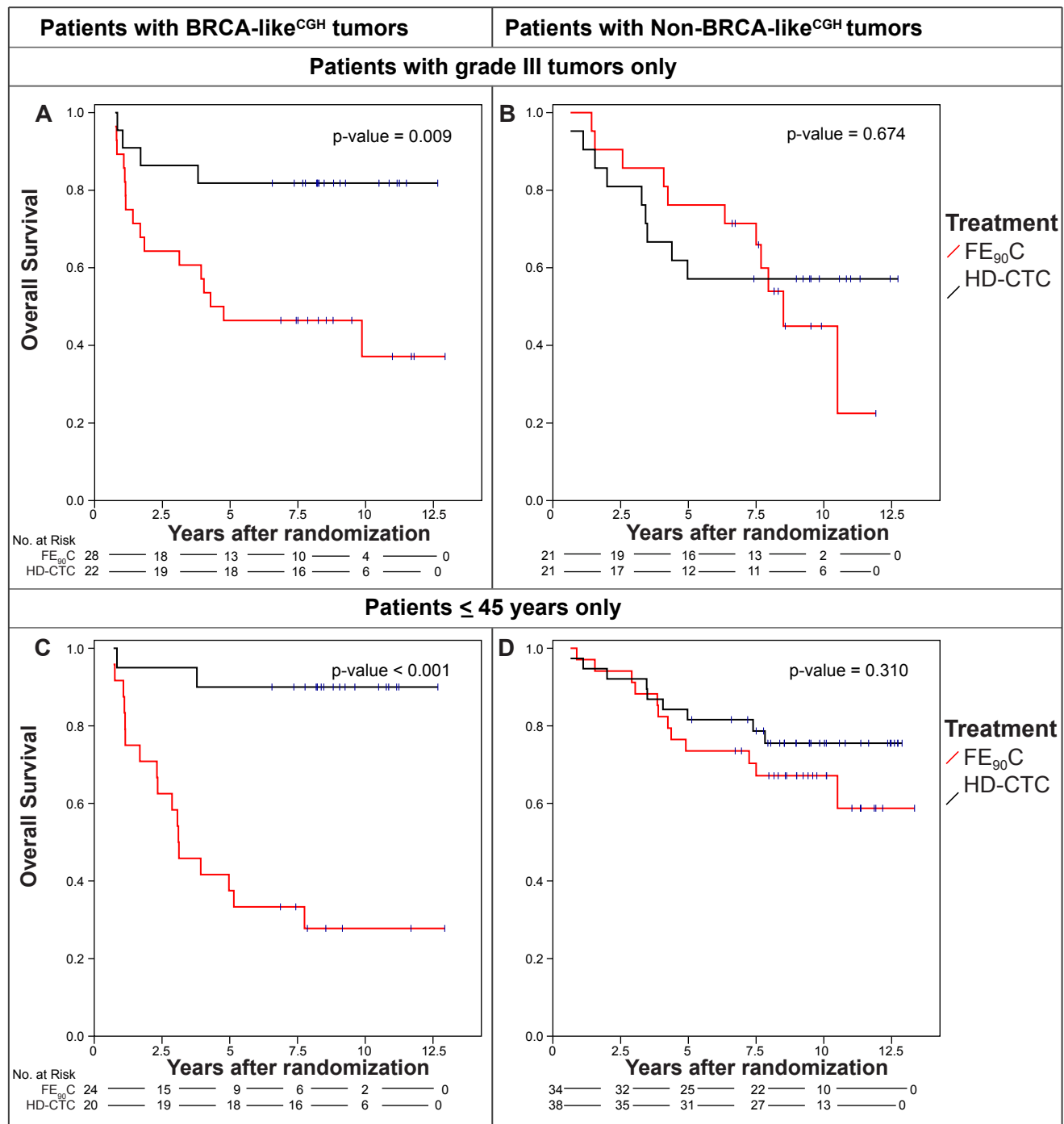

Supplement: Additional file 2: Figure S1 — Overview of histological patient characteristics and aCGH classification per patient. Figure S2. Association of the BRCA-like CGH status with recurrence-free survival after HD-CTC and conventional FE90C chemotherapy. Figure S3. Association of the BRCA1-like CGH, the BRCA2-like CGH and the non-BRCA-like CGH status with overall survival after HD-CTC and conventional FE90C chemotherapy. Figure S4. Association of the BRCA-like CGH and the non-BRCA-like CGH status with overall survival after HD-CTC and conventional FE90C chemotherapy in patients with grade III tumors and in patients younger than 45 years. [file bcr3655-S2.pdf]
